# Seeds of Change: exploring the transformative effects of seed priming in sustainable agriculture

**DOI:** 10.1111/ppl.70226

**Published:** 2025-04-29

**Authors:** Eva Cañizares, Luca Giovannini, Berivan Ozlem Gumus, Vasileios Fotopoulos, Raffaella Balestrini, Miguel González‐Guzmán, Vicent Arbona

**Affiliations:** ^1^ Dept. Biologia, Bioquímica i Ciències Naturals Universitat Jaume I Castelló de la Plana Spain; ^2^ Consiglio Nazionale delle Ricerche (CNR), Istituto per la Protezione Sostenibile delle Piante (IPSP) Torino Italy; ^3^ Consiglio Nazionale delle Ricerche, Institute of Biosciences and Bioresources (CNR‐IBBR) – Bari Italy; ^4^ Department of Agricultural Sciences, Biotechnology and Food Science Cyprus University of Technology Limassol Cyprus

## Abstract

The threats posed by climate change on agriculture at a global scale have fostered researchers to explore new and efficient strategies to ensure stable and safe food production. These new strategies must not only be efficient in reducing yield loss but also comply with environmental and consumer safety regulations, which particularly refer to restrictions to pesticide application as well as the implementation of genetically modified organisms, including CRISPR/Cas edited lines. Among other approaches, priming constitutes an easier and relatively cheaper strategy to cope with the effects of abiotic and biotic stresses by boosting plants' endogenous potential. Particularly, pre‐sowing seed priming has proven effective in improving germination and seedling establishment as well as tolerance to environmental and biotic factors throughout the plant's life cycle, exhibiting clear long‐lasting effects. This tolerance response to a wide range of adverse factors is associated with physiological, metabolic and genetic mechanisms and responses at the seed level and subsequently in the established plant. The genetic and epigenetic mechanisms enabling this tolerance response in plants and their subsequent generation, as a transgenerational effect, will be reviewed. Finally, the potential of the different seed priming approaches contributing to an ecologically and economically more sustainable agriculture will be discussed.

## 1 INTRODUCTION

As the climate crisis continues to alter historical temperature records and precipitation patterns across the globe, global agricultural productivity has slowed its growth over the past 50 years, leaving the current crop situation in a highly vulnerable state (IPCC, 2023). Studies show climate change is likely to affect crop yields in different ways depending on crop species and location (FAO, 2018). In regions like Africa and South Asia, where warming will exceed 2°C, crops like wheat and corn will suffer major yield losses (You et al., 2009; Pequeno et al., 2021). Mediterranean countries are amongst the most vulnerable to the devastating effects of climate change; they will face exacerbated challenges due to climate change in the coming decades. For instance, water availability is expected to decrease by 25% by 2050, while the frequency and intensity of severe climatic events are anticipated to increase, leading to consequences such as reduced crop yields and higher incidence of pests and diseases (MedECC, 2020). Changes in rainfall patterns could also lead to soil erosion, which is a major concern in many parts of the world, as heavy rainfall washes away topsoil and provokes nutrient depletion, which increases the risk of landslides and other natural disasters (IPCC, 2023). In addition to soil degradation and erosion, climate change may exacerbate the problem of soil salinization, particularly in irrigated areas, potentially leading to a reduction in the fertility of agricultural land (Eswar et al., 2021). Similarly, rising temperatures will increase evapotranspiration rates, exacerbating the problem of water scarcity, which may also affect already depleted soils. Moreover, higher temperatures will lead to changes in crop phenology and important shifts in reproductive events, with plants ripening earlier in the year, hence rising the incidence of pests and diseases (Moriondo et al., 2010). This will be particularly problematic in perennial fruit crops such as citrus, grapevine or olive trees (De Ollas et al., 2019). Taking it all together, the European Environment Agency has evaluated that climate change could reduce Mediterranean crop yields by 30% in the coming years (EEA, 2019).

In the last decades, several new strategies have been explored and tested in the quest for solutions to address the climatic challenges. Engineering crop varieties to enhance tolerance and/or resilience to adverse environmental conditions faces the restrictive EU regulations about genetically modified organisms (GMOs). That may potentially dissuade European breeders from embracing these innovative methods to create new cultivars (Zimny et al., 2019). Apart from the challenges posed by climate change, the overfertilization imposed to enhance soil and crop productivity has resulted in the uncalculated contamination of arable lands and groundwater sources, as highlighted in reports by the European Environmental Agency (EEA) in 2019 and the Mediterranean Experts on Climate and Environmental Change (MedECC) in 2020. Therefore, the exploration of sustainable and legally compliant strategies is imperative in navigating the complex nexus of agricultural innovation and environmental conservation.

For these reasons, farming and scientific research groups are now focusing on the development of environmentally friendly techniques that have been shown to improve survival, development, and production for most crops of economic interest in an agro‐ecological framework (Gallardo‐López et al., 2018; Wezel and Bellon, 2018; van der Ploeg, 2020). Priming is an environmentally friendly method that can be applied directly to seeds prior to sowing, thereby avoiding field or crop chemical applications and facilitating subsequent crop production (Srivastava et al., 2021). It also plays a crucial role in modern agriculture by offering multiple benefits, including disease and pest resistance, improved germination, enhanced nutrient availability, improved secondary metabolism profile and yield uniformity (Conrath et al., 2015; Devika et al., 2021; Gohari et al., 2024a). This review will focus on the current knowledge about the effects of seed priming, the different strategies employed and the mechanisms by which seed priming exerts its effects. Potential applications of these techniques in modern agriculture will also be highlighted.

## 2 PRIMING TECHNOLOGIES

In ancient Greece, several texts already report many attempts to improve seed germination; “Some even pre‐soak the seed of cucumber in milk or water to stimulate germination” (Theophrastus, D.H.P. Book VII, 1: 6); “pre‐soaking of cucumber seeds in water and honey” (Plinius, N.H. Book XIX, XXII: 64; Evenari 1984). More recently, the studies conducted by May et al. (1962) and Ells (1963) highlighted some key points in seed treatment leading to better seed germination, paving the way for the modern concept of seed priming. Hence, seed priming is a treatment where seeds are partially hydrated to trigger early metabolic processes, enhancing germination speed, uniformity, and stress tolerance. This method improves plant resilience by exposing seeds to factors like osmotic pressure, different compounds, or microorganisms, inducing a specific physiological state before germination (Ashraf & Foolad, 2005). This state, termed the “primed” state, allows plants to activate defense responses more rapidly and to a higher degree upon the occurrence of the threat, gaining prominence as a stress management practice by protecting plants from both biotic and abiotic stressors without significant impact on fitness (Conrath et al., 2006; Tanou et al., 2012; Jisha et al., 2013; Savvides et al., 2016). For instance, Heydecker et al. (1973) succeeded in improving the germinability of onion (*Allium cepa*) seeds using high molecular weight polyethylene glycol (PEG), applying it as a seed priming method. Since then, numerous articles have been published on the use of various priming agents not only to promote homogeneous germination and seedling establishment but also to improve plant performance under biotic and abiotic stress (Damalas et al., 2019; Adhikari et al., 2021; Ma et al., 2024; George et al., 2024).

*In planta* priming can be achieved by a mild level of any biotic or abiotic stress (primary stimulus) like pathogens, beneficial microbes, insects, chemical elicitors, which bring the plant into the priming phase, and it prepares the plant to respond better to a subsequent stress (effector). We talk about “cis‐priming” when the effector and the initial stimulus are the same. For example, it has been documented in Arabidopsis plants that non‐lethal heat treatment can confer acquired thermotolerance (Lämke & Bäurle, 2017), or that osmopriming with NaCl of Arabidopsis seeds conferred resistance to subsequent salt stress (Biswas et al., 2023). A significant amount of work has been reported supporting the “cis‐priming” mechanism, both involving abiotic (Charng et al., 2007; Ding et al., 2012; Sani et al., 2013; Hincha & Zuther 2014) or biotic stimuli (Frost et al., 2008; Döll et al., 2013; Helms et al., 2014). On the other hand, when the initial stimulus is different from the second stress or effector, we talk about “trans‐priming” (Hiker et al., 2016). For example, the positive response showed by the addition of exogenous chemical compounds against heat stress as effector or priming using a living organism, *Aspergillus fumigatus*, as initial stimulus versus the effects of water stress in wheat, has been documented by several authors (Campos‐Soriano et al., 2011; Xu et al., 2016; George et al., 2024). In addition, numerous works have described a tolerance mechanism referred to as “cross‐tolerance” that allows plants exposed to an initial mild stress or to beneficial microbes to acquire a systemic resistance to multiple stresses, even of high severity (Pastori & Foyer, 2002; Vallad & Goodman, 2004; Jung et al., 2012; Hossain et al., 2018; Katam et al., 2020; Tsaniklidis et al., 2020; Yoshida et al., 2022; Zeng et al., 2022).

Numerous seed treatments such as seed hardening, film coating, pelleting, and encrusting have been effective ways to improve seed handling, germination, and crop establishment in some cases (Tripathi et al., 2024). Seed hardening focuses on enhancing the seeds' resilience to stress after exposition to specific conditions (reduced moisture, lower temperatures, or nutrient limitations) to prepare them for harsher environments after germination (Farooq et al., 2006). Film coating consists of creating a thin polymer layer around seeds infused with nutrients, pesticides, or growth‐promoting agents, offering pest protection and even nutrient distribution while keeping the seed's original size and shape for easy planting (Pedrini et al., 2017). Pelleting covers seeds in a thicker layer of inert material, often clay, which can also contain slow‐release nutrients or pesticides and rounds out small or irregular seeds, making them easier to handle and sow precisely. Encrusting adds a moderate layer, enlarging seeds without fully altering their shape, balancing ease of planting with cost‐effectiveness. These methods prepare seeds for faster germination, better protection, and improved planting efficiency (Pedrini et al., 2017; Corbineau et al., 2023). However, seed priming involves soaking seeds in water or other solutions for a limited time to initiate metabolic processes, followed by drying to prevent germination.

From a farmer's perspective, on‐farm priming is often the preferred option for those with limited access to specialized facilities, as it provides flexibility and affordability while still delivering significant benefits for crop establishment (Carrillo‐Reche et al., 2018). On‐farm priming involves soaking seeds in water for a controlled period before sowing, allowing metabolic processes to initiate without full germination. It improves seedling vigor, promotes uniform emergence, and enhances resilience to drought and other abiotic stresses, particularly in rainfed agricultural systems. Additionally, on‐farm priming eliminates the need for specialized storage conditions, as seeds are sown immediately after treatment, reducing the risk of deterioration (Wojtyla et al., 2016). Traditional priming, on the other hand, is a well‐established method that involves pre‐treating seeds in controlled environments such as laboratories or commercial facilities (Tripathi et al., 2024). While this method requires more infrastructure and may be less accessible to smallholder farmers, it remains an effective way to enhance seed performance, particularly for large‐scale or commercial farming (Srivastava et al., 2021). Ultimately, priming—whether done on‐farm or through controlled methods—is a highly beneficial practice that improves crop productivity, making it a valuable tool for farmers across different scales and economic backgrounds (Carrillo‐Reche et al., 2018; Srivastava et al., 2021). The most common traditional seed‐priming technologies are hydro‐priming, osmo‐priming, chemo‐priming, hormo‐priming and bio‐priming (Table 1). Moreover, other novel techniques, such as hybrid priming and nanopriming, have emerged and are discussed later in this review (see sections 4.1 and 4.2, respectively).

**TABLE 1:** List of the most conventional seed priming techniques used in agronomy to improve germination percentage, plant efficiency and crop performance.

Technique	Description	Mecanisms	Crops	Benefits	References
**Hydro‐priming**	The process of soaking seeds in water for a predetermined duration to initiate physiological processes without germination.	‐Activation of enzymes (e.g., α‐amylase) ‐Enhanced mitochondrial respiration	*Zea mays* (maize), *Triticum aestivum* (wheat), *Glycine max* (soybean)	Enhances germination rates, uniformity, and seedling vigor.	Farooq et al., (2006); Ghassemi‐Golezani, K. (2008); Damalas et al., (2019); Adhikari et al., (2021)
**Osmo‐priming**	Soaking seeds in an osmotic solution (e.g., polyethylene glycol) to regulate water uptake and induce germination processes.	‐Induction of aquaporins for improved water transport ‐Upregulation of stress‐responsive genes (e.g., LEA proteins)	*Oryza sativa* (rice), *Solanum lycopersicum* (tomato), *Helianthus annuus* (sunflower)	Improves drought tolerance and promotes early seedling vigor.	Sani et al., (2013); Bourioug et al., (2020); Lei et al., (2021); Ma et al., (2024)
**Chemo‐priming**	Treatment of seeds with growth regulators or nutrient solutions to stimulate metabolic activity.	‐Enhanced germination enzymes activities ‐Increased secondary metabolites	*Helianthus annuus* (sunflower), *Lactuca sativa* (lettuce), *Cucumis sativus* (cucumber), *Valerianella locusta* (corn salad)	Enhances metabolic activity, germination speed, seedling establishment and stress tolerance.	Lavanya et al., (2018); El‐Serafy et al., (2021); Yang et al., (2022); Tripathi et al., (2024); Gohari et al., (2024a)
**Hormo‐priming**	Treating seeds with solutions containing phytohormones (e.g., auxins, cytokinins) to enhance germination and growth potential.	‐Modulation of hormonal balance to stimulate metabolic processes ‐Enhanced stress tolerance through hormonal signaling	*Oryza sativa* (rice), *Triticum aestivum* (wheat), *Vigna radiata* (mung bean)	Improves germination rates, seedling establishment, and stress resistance.	Farooq et al., (2013); Singh et al., (2020); Basit et al., (2022); Chakma et al., (2021)
**Bio‐priming**	Inoculating seeds with beneficial microorganisms or plant extracts to enhance growth and stress tolerance.	‐Induction of systemic resistance mechanisms ‐Improved nutrient mobilization.	*Brassica oleracea* (cabbage), *Capsicum annuum* (pepper), *Cucumis sativus* (cucumber)	Promotes disease resistance, improved growth, and plant health.	Wright et al., (2003); Banerjee & Roychoudhury, (2023); Irshad et al. (2023)

## 3 EFFECTS OF SEED PRIMING

Seed germination represents one of the most essential and critical physiological events in the plant life cycle. It is frequently subject to stress factors from the environment and biological agents that can cause uneven germination (Han et al., 2017). By exploring the interactions between seed germination processes and defense priming mechanisms, researchers can gain valuable insights into how seeds can be conditioned for improved germination performance (Biswas et al., 2023; Diya et al., 2024). This understanding could lead to innovative agricultural practices aimed at enhancing seed viability and resilience, particularly in the face of climate change, ultimately promoting crop productivity. To attain this objective, it is necessary to understand the effects of seed priming at the different stages of plant development.

### 3.1 Seed stage

**FIGURE 1:**
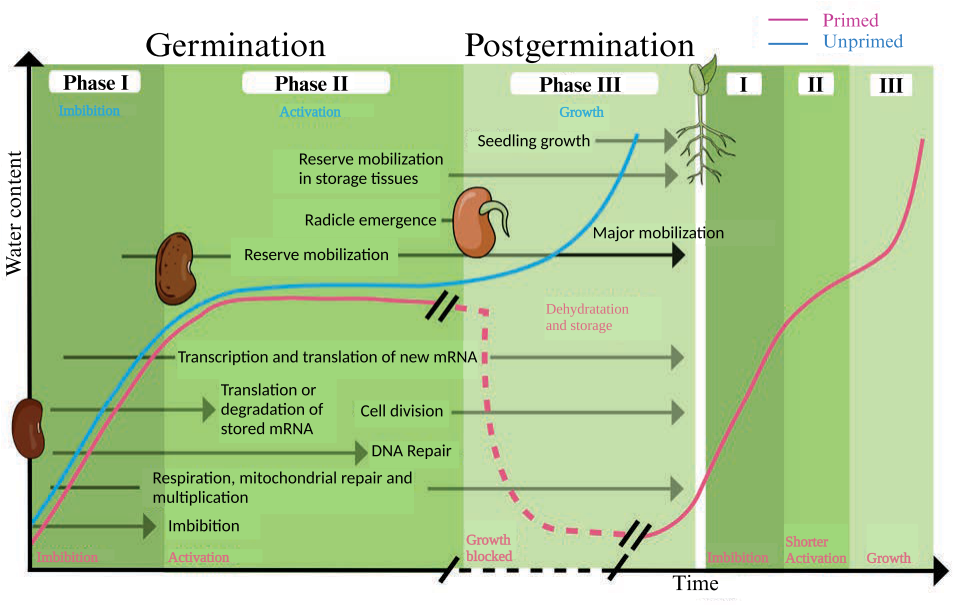
Physiological, biochemical and molecular events related to a normal germination process (blue) and a seed priming process during germination (red). Brackets show an alteration in the x‐axis scale subject on the nature of priming or the extent of storage of primed seeds. The curve displays a schematic time progress of water uptake. Modified from Nonogaki et al. (2010) and Corbineau et al. (2023). Created with Biorender.com.

The process of seed germination begins with the absorption of water and concludes with the emergence of the radicle (Farooq et al., 2009; Han et al., 2017; Johnson & Puthur, 2021). This absorption of water happens in three phases, each marked by specific physiological changes (Figure 1). Phase I involves a rapid uptake of water, triggering alterations in cell membranes and the reorganization of cell structure and molecules needed for cellular metabolism, which causes some cellular damage. This includes alteration of protein residues, loss of specific DNA sequences, breaking of DNA strands, and changes in DNA structure. Such protein residues comprehend the conversion of aspartyl residues of proteins to isoaspartyl, thus leading to increased sensitivity to aging and loss of seed vigor under germination conditions. During repair, some protein residues are converted back from isoaspartyl to aspartyl by the action of isoaspartyl methyltransferase (PIMT). This repair pathway is likely to work in concert with other anti‐aging pathways to actively eliminate deleterious protein products while some other genes responsible for DNA repair (ligases, poly‐ADP‐ribose polymerases, etc.) become more active, thus enabling a successful seedling establishment and strengthening plant proliferation in natural environments (Weitbrecht et al., 2011; Srivastava et al., 2021).

Seed priming treatment accelerates the initial stages of seed growth by overcoming any event of defective repair, like the gradual loss of telomeric sequences, DNA strand breakage, the loss of proper DNA confirmation and any other incidents produced by oxidative damage, which can cause cell death during germination (Figure 1; Devika et al., 2021; Srivastava et al., 2021). For example, the genes encoding the α‐ and β‐TYROSYL‐DNA PHOSPHODIESTERASE 1 from *Medicago truncatula* (*MtTdp1α* and *MtTdp1β*), two DNA repair enzymes, and the ones encoding distinct isoforms of DNA TOPOISOMERASE I (*MtTop1α* and *MtTop1β*), two DNA topology controlling enzymes, were found to be upregulated in seeds during imbibition in PEG (Balestrazzi et al., 2011). Moreover, seed priming activates DNA repair mechanisms and increases levels of antioxidants and osmoprotectants like superoxide dismutase (SOD), catalase (CAT), and peroxidase (POD), which contribute to balance the production of reactive oxygen species (ROS) levels [such as hydrogen peroxide (H_2_O_2_) and superoxide radicals (O_2_^●−^)], hence protecting the embryo from oxidative damage and promoting seedling growth (Farooq et al., 2017; Devika et al., 2021; Banerjee & Roychoudhury, 2023; Irshad et al., 2023; Diya et al., 2024). Biswas et al. (2023) reported that seed priming can induce the expression of 952 genes and 75 proteins in *Brassica napus* related to seed germination, DNA replication and repair, and cell cycle regulation, as well as the gene expression reprogramming of antioxidant genes (Figure 1). These responses have been shown to protect the primed seedlings from osmotic‐induced damage, resulting in enhanced growth and development compared to non‐primed seeds (Balestrazzi et al., 2011; Wojtyla et al., 2016; Biswas et al., 2023).

Phase II is characterized by a pause in water absorption while metabolic activities continue steadily, which prepares the groundwork for cell elongation and growth (Figure 1). It includes expression of genes related to respiration, reserve mobilization, osmotic adjustment, hormone regulation, sugar metabolism, cell wall development, antioxidant system, and protein turn‐over, although the period over which these phases take place is crop‐specific and varies significantly (Han et al., 2017). As shown in Hussain et al. (2016a), various species like maize, rice, tobacco, and chickpea (*Cicer arietinum*) exhibit heightened metabolic processes during this phase due to priming, leading to enhanced, rapid and uniform germination thanks to a reduction in the lag time of imbibition (Figure 1). Moreover, Han et al. (2017) have also suggested that priming triggers changes in the biochemical composition of seeds, resulting in a different metabolic state, which probably also affects seed germination and seedling growth.

During phase II, seeds also start synthesizing enzymes like α‐amylase, which breaks down stored starch reserves into simpler sugar forms, as well as proteases and lipases, which provide the necessary building blocks for growth and early development (Figure 1) (Varier et al., 2010; Paparella et al., 2015; Ju et al., 2019; Corbineau et al., 2023). It has also been shown that seed priming affects the levels of some compatible osmolytes, such as carbohydrates and carbohydrate derivatives (*e.g*. trehalose, mannitol or myo‐inositol), certain aminoacids (*e.g*. proline), and ternary ammonium compounds (*e.g*. glycine betaine; Cuin & Shabala, 2007), which help maintain the osmotic balance of cells with their surrounding environment without interfering with the biochemical activity of the cell (Cuin & Shabala, 2007). In chickpea, osmo‐priming resulted in increased activity of α‐amylase and sugar content during seed germination. Moreover, in those germinated seedlings, the CaCl_2_ seed priming enhanced trehalose and sucrose contents under optimal as well as chilling stress conditions (Farooq et al., 2017). As shown in Paparella et al. (2015), Selenium (Se)‐mediated seed priming resulted in an increased amount of soluble sugars in wheat (*Triticum spp*.) seeds, which have been postulated as a molecular signal playing a main function in the adjustment of plant water status, hence increasing drought tolerance, enhancing seed germination and radicle extension. Phase III involves again a rapid increase in water absorption by seeds, facilitating cell expansion, DNA replication, and cell division (Figure 1). Certain endosperm's cell wall remodeling proteins are triggered during this phase, such as expansins, which act in endosperm tissue destabilization, facilitating radicle emergence to start germination (Nonogaki et al., 2010; Srivastava et al., 2021; Corbineau et al., 2023).

Rapid seedling emergence is critical for successful crop establishment, particularly in water‐limited environments, and hydration‐dehydration produced by seed priming techniques has been shown to enhance this process by initiating early metabolic activity without full radicle emergence (Han et al., 2017; Srivastava et al., 2021; Biswas et al., 2023). These techniques enhance germination rate, synchronize emergence and improve vigor, making the crop more competitive with weeds and more efficient in using soil nutrients and water. In addition, by enhancing physiological and biochemical responses (Hussain et al., 2016a; Corbineau et al., 2023), primed seeds often develop greater tolerance to abiotic stresses such as drought and salinity. However, inappropriate dehydration after priming can compromise the viability of the seed, and storage conditions need to be carefully controlled; timely sowing is also required to maximize effectiveness, as the benefits of priming may diminish over time (Wojtyla et al., 2016). Despite these challenges, when optimized for specific crop species and environmental conditions, hydration‐dehydration priming or hydropriming remains a valuable tool for improving crop resilience and establishment (Worrall et al., 2011; Biswas et al., 2023).

Interestingly, several previous reports have shown that seed priming benefits can continue throughout the plant life cycle, even transferring to subsequent generations through a phenomenon known as “transgenerational priming memory” (Conrath, 2006; Tabassum et al., 2018). To explain this, two potential mechanisms have been proposed: one involves the accumulation of mitogen‐activated protein kinases (MPKs), while the other suggests that epigenetic changes in DNA methylation and histone modifications may serve as carriers of stress memories that trigger immune responses (Pastor et al., 2014; Conrath et al., 2015; Yang et al., 2022). Next, in this review, we will simplify the current understanding of seed‐priming molecular mechanisms by categorizing knowledge across different omics to elucidate the seed‐priming process across various species.

### 3.2 Effects of seed priming on plants

In plant vegetative tissues, the exposition to an external stimulus leads to the activation of molecular signals and metabolic pathways known as the post‐challenge priming phase, which leads to the accumulation of chemicals related to plant growth or the modulation of metabolism. This mechanism analyzed on different priming stimuli often shares a common subset of mechanisms that are then referred to as the “priming fingerprint”, as well as specific ones, like the activation of starch metabolism, based on the activities of various molecules like signaling proteins (receptors, kinases, phosphatases, etc.) and transcription factors, enzymes and plant hormones, but it might also involve epigenetic changes that affect the expression of defense genes that contribute to set and keep the primed state (Mauch‐Mani et al., 2017; Lämke & Bäurle, 2017; Tiwari et al., 2022).

**FIGURE 2:**
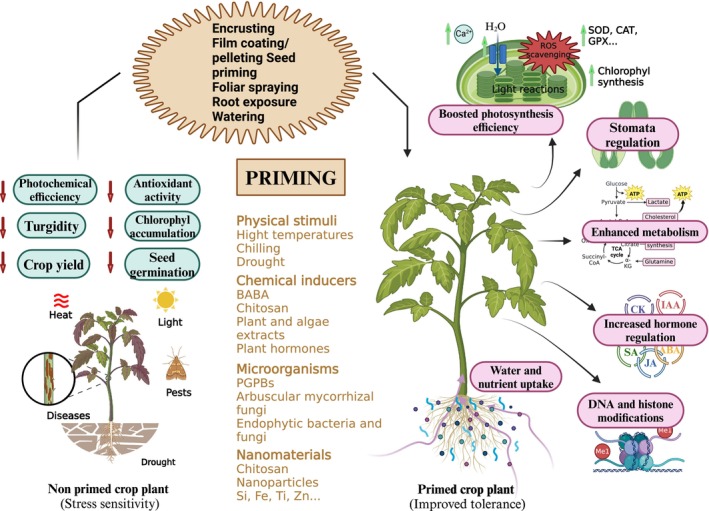
Schematic overview outlining the mechanisms by which primed plants derived from different techniques develop tolerance to environmental stress. Abbreviations: Superoxide dismutase (SOD), Catalase (CAT), Glutathione peroxidase (GPX), Reactive oxygen species (ROS), Plant Growth‐Promoting Rhizobacteria (PGPR), Cytokinins (CK), Salicylic acid (SA), Indoleacetic acid (IAA), Jasmonic acid (JA), and Abscisic acid (ABA). Created with Biorender.com.

Therefore, seed priming not only acts as a powerful agronomic strategy for enhancing germination but also improves seedling vigor, nutrient efficiency, and increases stress resilience, leading to higher yields, better grain quality, and improved crop resilience under adverse conditions (Bourioug et al., 2020; Chakma et al., 2021; Gohari et al., 2024a). By optimizing metabolic pathways, strengthening antioxidant defenses, improving water and nutrient uptake, and stabilizing hormonal balance, priming treatments ensure sustainable crop production under changing climatic conditions (Conrath et al., 2015; van der Ploeg, 2020). The following paragraphs unravel these changes, which manifest themselves at different levels (Figure 2):

#### 3.2.1 Physiological responses associated with seed priming

The retention and retrieval of priming stress memory are critical when prolonged intervals separate stress events. In their case, plants need to rely on alternate molecular mechanisms for information storage. Chromatin organization, transcriptional regulation, post‐translational modifications, phytohormonal signaling, and metabolite dynamics play pivotal roles in mediating this process, facilitating adaptive plant stress responses (Table 2) (Pastor et al., 2014; Hilker & Schmülling, 2019). For example, the resistance to downy mildew disease shown by plants exposed to lipopolysaccharide (LPS) elicitors seem to be related to nitric oxide (NO) production, defense genes activation such as pathogenesis‐related proteins (PRs), changes in the cell wall, and by the overproduction of hydrogen peroxide (H_2_O_2_; Lavanya et al.,^2018^). Similarly, during flooding stress, rice plants derived from SE‐ and SA‐primed seeds exhibited altered exp ression of genes involved in photosynthesis (*e.g. Chl a/b*‐binding protein, *RBCS4*), cell death (putative NBS‐LRR‐like protein), aerenchyma formation, and adaptation to submerged environments. Key ethylene response factors (ERFs), such as *ERF47*, *ERF108*, *ERF35*, *ERF20*, and *ERF79*, were notably upregulated due to seed priming, enhancing the submergence tolerance of seedlings derived from primed seeds (Hussain et al., 2016b).

**TABLE 2:** Classification of priming treatments according to their respective target cell pathway and the molecular factors involved.

Type	Target molecule(s)	Priming agent	Factors involved	Physiological effect	References
**Hormonal**	Accumulation of different plant hormones	Chemical (β‐aminobutyric acid, BABA), biotic elicitors	JA, ABA and SA hormonal pathways; IAA pathway, particularly AUX/*IAA* genes	Tolerance to drought stress; induction of defenses against biotic threats; high‐light protection	Pastor et al., 2014; Barnawal et al., 2017
**Enzyme activity**	Increased activity of antioxidant enzymes	Biotic elicitors (eg. *Peanibacillus sp.; Trichoderma asperellum*)	Superoxide Dismutases (SOD) Peroxidases (POD) Catalase (CAT)	Tolerance to drought stress and osmotic stress	Mauch‐Mani et al., 2017
**Metabolism**	Sugars, Lipids, Green Leaf Volatiles (GLVs) production	Osmopriming; Chemical (BABA); biotic elicitors (*T. asperellum*)	Oxylipins, JA, C6‐alcohols, C6‐aldehydes, and C6‐acetates	stress‐signaling molecules against heat or chilling stress, protection against herbivory	Farooq et al., 2017; Yang et al., 2022
**Epigenetic Mechanisms**	DNA/chromatin marks: Histone modification, DNA methylation	Osmopriming	H3K4me3 mark within *P5CS1* promoter	Salt stress tolerance	Biswas et al., 2023
**Transcriptional Memory**	Transcription Factors (TFs) induction	Chemical (acibenzolar S‐methyl, BABA)	MYB; HsfA2; WRKY	Thermotolerance	Jaskiewicz et al., (2010); Yang et al., 2022
**Post‐translational Regulation**	Protein kinases MAPKs, Ca^2+^‐sensing protein kinases (CPKs) phosphorylation	Arbuscular Mycorrhizal Fungi (AMF); Pathogen‐Associated Molecular Patterns (PAMPs)	*MPK3, MPK9*	Osmotic stress tolerance	Bender et al., (2017); Hake and Romeis, (2018)

In this respect, the growth promotion observed in seeds from lettuce exposed to salt but previously inoculated with *Azospirillum brasilense* may be attributed to a higher net assimilation rate, which reflects the net result of carbon gain (photosynthesis) and carbon losses (respiration, exudation, volatile compound emission) expressed per unit leaf area (Fasciglione et al., 2015). Other species, such as Arabidopsis, have developed salt tolerance by accumulating high levels of sugars, amino acids, and intermediary metabolites from the shikimate pathway, including coniferin. Additionally, they exhibit increased lignin content, resulting in significantly thicker cell walls. These findings highlight the importance of both biochemical adjustments and physical modifications in adapting to high‐salinity conditions (Chun et al., 2018). Accordingly, *Azospirillum*‐inoculated wheat plants showed growth improvement under saline conditions due to enhancement in photosynthetic pigment and solute concentrations as well as N assimilation, hence adjusting the leaf osmotic potential (Nia et al., 2012). These changes also influenced other defense mechanisms, such as the production of enzymes and proteins against pathogens, such as hydroxyproline‐rich glycoproteins (HRGPs), which are part of the plant cell wall and play a crucial role in enhancing the plant resistance against downy mildew disease (Lavanya et al., 2018).

Similarly, seeds of chili pepper plants (*Capsicum anuum* L.) treated with the bacterium *Peanibacillus dendritiformis* or beneficial fungi *Trichoderma asperellum* and *Trichoderma harzianum* showed less disease‐related damage when infected with the harmful fungus *Colletotrichum truncatum*. These increased the activity of several antioxidant enzymes [like superoxide dismutase (SOD), catalase (CAT), and peroxidase (POD)] that help plants to resist and overcome stress by lowering the production of ROS related to the disease progression (Figure 2), (Mauch‐Mani et al., 2017; Tripathi et al., 2024). In addition, the total amount of certain protective compounds in the plants, namely phenolics, increased after seed priming treatment. This increase was greater when the plants were challenged by the fungus *Colletotrichum truncatum*, contributing to making plant cell walls thicker by lignin deposition, thus making plants more resistant to the disease. Furthermore, primed plants showed increased levels of chlorophyll, which correlated with improved photosynthetic activity (Figure 2; Yadav et al., 2021).

At the molecular level, stimulus perception and downstream cellular immune responses are rapidly linked by sequential phosphorylation events, which play a key role in the rapid activation of phosphorylation‐dependent defense mechanisms (Hake & Romeis, 2018). In this work, priming led to increased expression of the *MPK3* and *MPK6* genes, as well as the accumulation of their inactive forms (unphosphorylated kinases). Hence, effector stress triggers the rapid activation and subsequent enhancement of the MPK‐dependent signaling, leading to improved defense responses of seed‐primed plants, which is supported by an impaired response in *mpk3* and *mpk6* mutant lines. On the other hand, Mauch‐Mani et al. (2017) have reported that pathogen‐associated molecular patterns (PAMPs), insect feeding, and arbuscular mycorrhizal fungi (AMF) transiently impact calcium levels during the priming phase. Furthermore, genetic and Ca^2+^ imaging studies in guard cells have shown that certain stimuli, such as ABA and CO_2_, increase the Ca^2+^ sensitivity of stomatal closing responses, indicating that Ca^2+^‐dependent pathways for stomatal closure can also be primed for activation. In Bender et al. (2017), it was proposed that the phosphorylation of Ca^2+^ sensors, particularly Ca^2+^‐dependent Protein Kinases (CPK) autophosphorylation, may serve as a biochemical marker of the primed state in Ca^2+^‐mediated signaling pathways. Moreover, the increase in cytosolic Ca^2+^ also triggers ion fluxes across the membranes, which leads to membrane depolarization. Interestingly, membrane depolarization triggers electrical signaling that can transmit the local perception of wounding to undamaged leaves and activate JA signaling in those leaves (Hake & Romeis, 2019).

#### 3.2.2 Influence of priming on hormonal responses and regulation

Several studies indicate that primed plants accumulate precursors of conjugated or inactive forms of defense‐related hormones, which are rapidly converted into their active forms, helping the plant respond faster to the effector stress (Table 2) (Worrall et al., 2011; Balmer et al., 2015; Mauch‐Mani et al., 2017; Hilker and Schmülling, 2019). For instance, tobacco plants treated with BABA or exposed to pathogenic bacteria (*e.g. Pseudomonas syringae*) partly metabolized the SA into SA 2‐O‐beta‐D‐glucose (SAG) or salicylic acid glucose ester (SGE), which can be rapidly hydrolyzed to SA when the plant is challenged by the effector stress (Pastor et al., 2014).

In maize, dual seed priming combining SA and H_2_O_2_ upregulated the expression of GA biosynthetic genes like *ZmGA20ox1* and *ZmGA3ox2*, and downregulated the GA catabolism gene *ZmGA2ox1*, while promoting the expression of the ABA catabolism gene *ZmCYP707A2*. In addition, the GA signaling mediators *ZmGID1* and *ZmGID2* were upregulated, leading to the promotion of the germination of maize seeds subjected to chilling (Li et al., 2017). The activation of some GA‐biosynthetic genes (*GA20OX1*, *GA3OX1*) and increased production of active GAs were observed also in tomato seeds subjected to NaCl‐priming, which accelerated seed germination (Nakaune et al., 2012; Biswas et al., 2023; Diya et al., 2024). In cucumber (*Cucumis sativus*), seed priming with benzothiadiazole (BTH) combined with biologically active compounds such as surfactin, iturin, and fengycin or heat‐stable metabolites and bacterial cyclodipeptides (cyclo(L‐Leu‐L‐Pro)) isolated from root‐associated *Bacillus spp*., induced disease resistance by boosting the plant immunity, inducing the expression of resistance marker genes related to SA, ethylene, and jasmonic acid (JA) signalling, resulting in increased mortality of the insect pest *Spodoptera litura* (Song et al., 2017). In wild tobacco (*Nicotiana benthamiana*) plants pre‐treated with a polypeptide extract of the dry mycelium of *Penicillium chrysogenum* (PDMP) did not induce a direct response but induced a defense response after tobacco mosaic virus (TMV) infection, helping plants to rapidly mount a resistance response after TMV challenge such as the callose deposition around plasmodesmata, which was associated to an enhanced ABA biosynthetic pathway (Li et al., 2021).

Biopriming of wheat plants with growth‐promoting bacterial strains of *Dietzia natronolimnaea*, *Arthrobacter protophormiae* and *Bacillus subtilis* modulated the auxin signaling pathway through the degradation of different AUX/*IAA* gene family of auxin response repressors, promoting the activation of *ARF* transcription factors and the subsequent expression of auxin responsive genes (Barnawal et al., 2017). In addition, Barnawal et al. (2017) reported that both salt and drought induce the upregulation of these genes, but previous inoculation with PGPR at the seedling stage led to its downregulation. Furthermore, plants inoculated only with the bacteria *D. natronolimnaea* exhibited protection from salt stress by modulating the expression of *TaCTR1* and *TaDREB2* genes, which enhanced the IAA level and reduced ABA and 1‐aminocyclopropane‐1‐carboxylate (ACC) content, an intermediate in ethylene biosynthesis (Barnawal et al., 2017; Mitra et al., 2021). In several species, like maize, rice and *Camelina sativa*, it has also been reported that some bacteria (like *Pseudomonas migulae*, *Rahnella aquatilis* and *Achromobacter sp*.) can increase the production and activity of ACC‐deaminase, which hydrolyzes ACC to ammonia and α‐ketobutyrate and lowers the level of ethylene, a phytohormone that plays a dual role in plants' survival under abiotic stresses, acting both positively and negatively. In appropriate amounts, ethylene supports key processes such as seed germination and root hair development, which are vital for nutrient and water absorption. However, under salt stress, elevated ethylene levels can negatively impact seed germination, plant growth, and overall yield (Tiwari et al., 2020; Mishra et al., 2021).

#### 3.2.3 Metabolic responses associated with seed priming

The modulation of plant metabolism to accumulate central and/or specialized metabolites (such as amino acids and phytoalexins) is a well‐known mechanism to prepare plants to a new threat. As summarized by Pastor et al. (2014), *A. thaliana* plants primed with BABA showed potentiation of the tricarboxylic acid (TCA) cycle with a strong induction of several compounds like citrate/isocitrate, S‐malate, 2‐oxoglutarate and fumarate. Moreover, *Pseudomonas syringae* priming treatment of Arabidopsis produces the accumulation of IAA via the Trp pathway through the synthesis of indole‐3‐pyruvic acid (IPA) and indole‐3‐acetamide (IAM). Conversely, BABA seems to use a Trp‐independent pathway since it produces a depletion of the levels of Trp to produce IAA as well as other conjugates (indole‐3‐acetyl‐L‐Ala, IALA) or derivatives of indol‐3‐carboxylates (indole‐3‐carboxylic acid methyl ester, I3CAME; Pastor et al., 2014). Similarly, in *Melissa officinalis* plants, seed priming with nano‐silicon (nSi) particles and two bacterial isolates (*Pseudomonas fluorescens* or *Pseudomonas putida*) led to significant increases in the levels of key essential oil metabolites, including citral, neral, geranial, and geranyl acetate. These changes suggest enhanced metabolic activity following the combined priming treatments. (Hatami et al., 2021). Furthermore, *Valerianella locusta* seeds primed with chitosan‐melatonin nanocomposites resulted in seedlings showing increased tolerance to salt stress with several polyphenolic compounds being induced, such as chlorogenic acid, naringine, o‐coumaric acid and catechin hydrate (Gohari et al., 2024a). Interestingly, treating grape berries with BABA helps them resist *Botrytis cinerea* infection and alters their sugar and phenylpropanoid levels. High doses of BABA boost genes related to phenylpropanoid production through the induction of the grapevine transcription factor called *VvMYB44* (Yang et al., 2022), contributing to the particular process known as BABA‐induced resistance (BABA‐IR), which is associated with an augmented capacity to express basal defense responses in a wide range of plants (Mauch‐Mani et al., 2017; Hilker and Schmülling, 2019).

In chickpea seed, osmopriming increased α‐amylase activity and sugar contents, including trehalose; this response was accompanied by an increase in antioxidant capacity, which protected against oxidative damage and helped to maintain carbon assimilation, hence contributing to seedlings' growth and development under both optimal and chilling stress conditions (Farooq et al., 2017). The fungus *Trichoderma asperellum* has been shown to activate defense responses in *Arabidopsis thaliana* against the virulent pathogen *Pseudomonas syringae* without significantly altering gene expression during the priming stage. At the metabolic level, there was an accumulation of amino acid precursors linked to plant defense, especially for indolic glucosinolate biosynthesis. Additionally, some compounds, like pipecolic acid, accumulated along with changes in leucine, valine, isoleucine, methionine, and glutamine levels. Changes in sugar content and levels of intermediates of the citric acid cycle and polyamine were also noted (Balmer et al., 2015). Moreover, the endophytic fungi *Aspergillus fumigatus* was used in George et al. (2024) to evaluate the cost‐effectiveness of enhancing the physiological and molecular drought resistance of various wheat cultivars after applying the seed biopriming. At the metabolic level, results indicated that plants bioprimed with *A. fumigatus* maintained higher levels of carotenoids and chlorophylls but exhibited significantly lower phenol content compared to those grown from unprimed seeds under drought conditions.

#### 3.2.4 Epigenetic changes linked to seed priming

Epigenetic mechanisms play a crucial role in enhancing plants' ability to respond to environmental stresses. Through processes such as DNA methylation and histone modification, priming can induce lasting changes in gene expression, “epigenetic memory”, that enhance stress tolerance; the resulting epigenetic modifications are decisive for the effectiveness of seed priming (Table 2) (Danker et al., 2007). Additionally, these epigenetic changes can be passed down to future generations, potentially promoting adaptive traits. Understanding the role of epigenetics in seed priming opens new avenues for the development of strategies enhancing crop resilience and productivity in the face of environmental challenges. Exploring epigenetics could help uncover the connection between priming effects and stress memory, as epigenetics governs inheritable changes in gene expression not associated to nucleotide sequence (Conrath et al., 2015).

The first thing to consider is the chromatin structure since it is crucial for regulating gene expression. The basic unit of chromatin, the nucleosome, consists of 147 base pairs of DNA wrapped around a core of histone proteins (H2A, H2B, H3, and H4). Histones undergo various modifications, such as (i) acetylation, which is linked to active genes by reducing the interaction between histones and DNA and attracting proteins that promote transcription, or (ii) methylation, which is more complex since methylation of specific lysine residues on histones H3 and H4 can either activate or repress transcription (Danker et al., 2007; Jaskiewicz et al., 2010). In addition, we must contemplate that mechanisms regulating the enzyme activity and subsequent changes in metabolism via modulating the transcript abundance and/or protein level are short‐lived compared with the DNA methylation and histone modification, which are long‐lasting processes (Hilker and Schmülling, 2019).

To test the hypothesis that primed genes might be activated faster due to histone changes, Jaskiewicz et al. (2010) studied several Arabidopsis genes that encode WRKY transcription factors (WRKY29, WRKY6, and WRKY53) after priming with acibenzolar S‐methyl, an analog of SA (Table 2). They found a significant increase in WRKY gene expression of primed plants when plants were stressed with pathogens and this upregulation was associated with changes in histone modification patterns. On the other hand, heat‐stress priming activates a transcription factor called HsfA2, which puts a H3K4me mark on certain genes related to heat stress‐memory such as APX2 (ASCORBATE PEROXIDASE 2) and HSP21 (HEAT SHOCK PROTEIN 21), contributing to the acquisition of thermotolerance (Lämke & Bäurle, 2017). Moreover, cold‐primed plants also show changes in gene activity related with increased histone acetylation and changes in histone variants like H2A.Z; still, the direct role of H2A.Z in cold‐stress memory is not clear yet (Srivastava et al., 2021). Another interesting result came from the osmopriming of Arabidopsis seeds with NaCl that increased plant resistance to subsequent salt stress (Biswas et al., 2023). After the whole genome analysis, authors found regions enriched in H3K4me3 or H3K27me3 in root tissues of primed plants, which were retained through subsequent mitosis in primed plants after salt stress. Besides, genome‐wide reprogramming of H3K4me3 and H3K27me3 also revealed that H3K4me3 acted as a marker for salt‐stress memory response on the gene encoding for D1‐PYRROLINE‐5‐CARBOXYLATE SYNTHETASE 1 (*P5CS1*), a rate‐limiting enzyme in proline biosynthesis pathway. An increase in *P5CS1* gene expression during priming led to H3K4me3 retention and transcriptional induction during the recovery stage after plants faced salt stress. A subsequent salt exposure further induced *P5CS1* expression, leading to increased proline accumulation and improved salt tolerance (Biswas et al., 2023).

This transgenerational epigenetic memory is characterized mechanistically not only by histone modifications but also by other chromatin changes such as DNA methylation, histone tail modifications, chromatin remodeling or stalled RNA polymerase II phosphorylated at its tail and bound to certain genes playing part in the coordinated changes in gene expression patterns that support memory responses (Danker et al., 2007; Chakraborti et al., 2021). The chromatin memory formed via methylation and histone modifications during seed priming is likely preserved through feedback mechanisms and cytosolic allocation during cellular divisions. This process promotes transgenerational plasticity, which is further enhanced by epigenetic modifications and signaling pathways activated during various stages of plant development (Biswas et al., 2023). The transgenerational mechanism for pathogen resistance was investigated in Arabidopsis plants primed with BABA or avirulent *P. syringae* strain (Slaughter et al., 2011). Their descendants showed a faster and higher accumulation of transcripts of defense‐related genes (SA‐dependent genes *PR1, PR2*, and *PR5*) in the SA signaling pathway due to changes in DNA cytosine methylation, enhanced disease resistance upon challenge with a virulent *P. syringae* strain or the pathogen oomycete *Hyaloperonospora arabidopsidis* (Slaughter et al., 2011).

## 4 FUTURE PERSPECTIVES

### 4.1 Hybrid priming

The application of multiple priming agents, including combined treatments, has been shown to enhance plant growth under various stress conditions. For instance, sunflower seedlings subjected to both hydro‐priming and osmo‐priming exhibited increased photosynthetic efficiency, higher quantum yield of photosystem II, improved stomatal conductance, enhanced evapotranspiration and increased grain yield and oil content, as compared to non‐primed controls, under both normal and stress conditions. These findings suggest that both hydro‐priming and osmo‐priming effectively improve the germination and growth performance of dormant sunflower seeds (Bourioug et al., 2020). Tabassum et al. (2018) showed that the progeny of drought‐stressed and osmo‐primed wheat plants exhibited better performance and stress tolerance under subsequent drought stress in several traits like leaf area, grain yield, chlorophyll contents, accumulation of phenolics, proline, glycine betaine, total soluble proteins, relative water contents, osmotic and pressure potentials, stability of the cell membrane, and grain Zn, Mn and B contents, while they had decreased malondialdehyde contents under drought stress, displaying an improvement in wheat plants performance.

Mechanical seed priming has been used to promote seedling development and vigor. Physical treatments such as pre‐sowing soaking, heating, and chilling were effectively combined to enhance seed vigor in both coarse and fine rice varieties. Soaking integrated with heating in fine rice showed higher values for final germination percentage, germination index, root and shoot length, as well as fresh and dry weights of seedlings, compared to other treatments, including the control (Farooq et al., 2006). Seed priming with nSi particles, combined with seedling inoculation using *Pseudomonas* strains, significantly enhanced both primary and secondary metabolite contents in lemon balm plants, improving phenolic content, essential oil yield, and all major constituents—except thymol—such as neral, geranial, and geranyl acetate, compared to the control. Additionally, these treatment combinations boosted the free radical scavenging activities of plant extracts more than the individual treatments or untreated controls. Additionally, these treatments induced various morphological and physiological mechanisms, such as the development of a more efficient root system, which improved nutrient uptake (Figure 2) (Hatami et al., 2021). This multifaceted approach leverages the synergistic effects of different priming agents, which can activate various physiological and biochemical pathways within the plant. Recent studies using hybrid priming to detect enhanced stress memory have shown promising results. For example, Garcia et al. (2021) observed changes in tomato gene expression at the transcriptional level, including a decrease in *SlNCED2* (ABA biosynthesis) and an increase in *SlDELLA* (GA regulation) expression after hydro‐electrostatic hybrid seed priming. This is in line with results reported by Li et al. (2017), where priming with SA and H₂O₂ synergistically enhanced seed vigour, stress resistance and seedling quality under chilling conditions by integrating reactive oxygen species regulation, carbohydrate metabolism, antioxidant defence and hormonal balance through gene up‐regulation (*e.g. ZmPAL, ZmSnRK2.1, ZmCPK11*). However, the epigenetic mechanisms behind hybrid priming, such as DNA methylation and histone modifications, remain unclear and require further investigation.

### 4.2 Nanopriming

Nanomaterials are substances with structures at the nanoscale (1 to 100 nm) that can exist in liquid or solid states (Buzea et al., 2007; Chung et al., 2016). These materials are gaining attention as seed priming agents to enhance germination, growth, stress tolerance and plant productivity by improving nutrient uptake, mitigating environmental stresses, and enhancing water retention (Kah et al., 2019; Mittal et al., 2020; Ioannou et al., 2020; Hatami et al., 2021). These include metal nanoparticles (*e.g*., silver, gold) and carbon‐based materials (*e.g*., graphene, carbon dots), as well as nanocarriers for efficient nutrient delivery (Mittal et al., 2020; Hatami et al., 2021). However, their use raises safety and regulatory concerns, necessitating careful evaluation of their long‐term effects on health and the environment. Given their potential to revolutionize agricultural practices, we will discuss the various types of nanomaterials, their mechanisms of action, and their implications for future seed priming methodologies in the following lines.

In the nano‐priming method, nanoparticles (NPs) can cross biological barriers and penetrate the seed coat, facilitating water uptake or remain retained on the seed surface instead (Pereira et al., 2021) but activating metabolic responses occuring in the early stages of germination, leading to an increase in seed germination rate (García‐Gómez & Fernández, 2019). Nanopriming with various nanomaterials has demonstrated significant enhancements in seed germination and seedling growth characteristics for a range of plant species, although the debate on the sustainability of nanomaterials is still alive (Chandrasekaran et al., 2020; Shelar et al., 2021; Amritha et al., 2021). Titanium oxide (TiO_2_), silicon dioxide (SiO_2_), Cu/CuO/Cu (OH)_2_, iron/iron oxides (Fe/FeOx), zinc/zinc oxide (Zn/ZnO), silver (Ag), gold (Au), NPs of biochar, NPs of lignin and cerium oxide (CeO_2_) NPs have been used to facilitate plant growth and development (Gottschalk et al., 2009; Pradas del Real et al., 2016; Kah et al., 2019). For example, the use of silica NPs has been shown to increase germination rates in tomato and maize (Singh et al., 2024). Furthermore, priming with zinc oxide nanoparticles has led to notable improvements in morpho‐physiological traits and antioxidant capacities in lupine (Zaki et al., 2023). Similarly, foliar application of ZnO NPs improved drought tolerance in *Dracocephalum kotschyi* plants by significantly enhancing the activity of antioxidant enzymes, including SOD and POD (Karimian & Samiei, 2023). They also promote chlorophyll content (Chl *a*/*b*) and carotenoids. On a metabolic scale, they modulate carbohydrate metabolism through carbonic anhydrase, accumulating proline, and pathways involved in protein biosynthesis. Additionally, the application of ZnO has been shown to enhance the activity of α‐amylase and β‐amylase in seeds, as well as total amylase in seedlings, thereby supporting improved seed germination and overall plant vigor (Karimian & Samiei, 2023). Nanopriming of seeds has also shown significant potential to modulate gene expression and enhance plant resistance to both abiotic and biotic stresses. For example, transcriptomic analyses of cotton seeds primed with poly(acrylic acid)‐coated cerium oxide nanoparticles revealed extensive gene expression changes, including *CAX1* (calcium transport), *PRX* (ROS detoxification), *GST* (antioxidant defense), and *TPS* (terpene synthesis), enhancing ion balance, oxidative stress response, and salinity tolerance in seedling roots (An et al., 2020). These findings highlight the complex molecular mechanisms by which nanoparticles also influence transcriptional, translational, and epigenetic regulation, ultimately activating stress memory mechanisms and optimizing plant responses to environmental challenges (Mittal et al., 2020). While the precise pathways remain partially elucidated, further research is essential to fully understand how nanoparticles act as signaling molecules or cofactors, fine‐tuning phytohormone regulation and stress response pathways. This approach offers promising strategies to enhance plant growth, improve stress resilience, and strengthen long‐term defense mechanisms (Pereira et al., 2021).

### 4.3 Biostimulants

Biostimulants as biopriming agents play a crucial role in boosting plant resilience to various abiotic and biotic stresses while enhancing resource use efficiency. A wide range of biological and natural chemical components such as plant growth‐promoting bacteria (PGPB), humic acids, natural antioxidants and polyamines, phytohormones and even raw materials such as algae extracts are being used to formulate innovative biostimulants (Ebeed et al., 2017; Nadeem et al., 2022). In recent years, the innovative ecologically friendly strategy of synthesizing biocompatible NPs enhanced the effectiveness of natural priming agents, promoted faster germination, improved stress resilience, and enhanced seedling growth (Pereira et al., 2021; Shelar et al., 2021; Tripathi et al., 2024). More recent advances include the functionalization of nanomaterials with chemical priming agents, whereby the nanomaterials act as smart carriers that further improve the activity of the priming agents and lower the dose needed (Gohari et al., 2024b).

Biostimulants are increasingly recognized for their role in enhancing plant growth and stress resistance by continuously stimulating physiological and metabolic processes, which improve plant health over time (Basit et al., 2022; Nadeem et al., 2022). In contrast, priming agents equip plants with a heightened defense capacity, enabling a faster and more robust response to environmental challenges. Such biostimulants can prepare plants to respond more effectively to environmental stresses by inducing changes in gene expression without altering the underlying DNA sequence. One notable example involves the application of a Bacillus‐based biostimulant to maize plants (Lephatsi et al., 2022). This treatment induced DNA hypermethylation, which correlated with the altered expression of key stress‐responsive genes (*e.g. DREB2A*, *P5CS*, *PAL* and *FSNII*), leading to improved drought tolerance and enhanced growth (Lephatsi et al., 2022). In that sense, Biondi et al. (2022) showed a differentially modulated ABA metabolism in quinoa (*Chenopodium quinoa*) plants derived from seeds primed with polyamines: spermidine downregulated NCED expression, whereas spermine upregulated it under salt stress conditions, potentially enhancing saline tolerance. Similarly, the use of lipopolysaccharides (LPS) for pearl millet seed priming enhanced plant immunity against *Sclerospora graminicola* by activating key defense genes (*e.g. PAL*, *POX*, *HRGP* and *PR‐1* or *PR‐5*) involved in stress response and pathogen resistance leading to a more effective immune response (Lavanya et al., 2018).

## 5 CONCLUDING REMARKS

At present, there is a gap in scientific knowledge regarding the precise molecular mechanisms behind the beneficial effects of seed priming, which limits our comprehensive understanding of the influence of seed priming at the whole plant level. Despite these limitations, employing a variety of priming strategies has been demonstrated to be a promising strategy to promote plant tolerance to multiple environmental stresses. This is particularly relevant for crop production in regions where climate change is exacerbating water scarcity, increasing occurrence of periods with extreme temperatures, and soil degradation. By increasing plant tolerance to these stresses, priming strategies not only contribute to ecological stability but also have significant economic implications. As traditional farming inputs like fertilizers and pesticides become costlier and less sustainable, seed priming offers an economically viable alternative to reduce input dependency while maintaining or even enhancing yields. Moreover, seed priming aligns well with the goals of sustainable agriculture, as it supports a reduction in agrochemical inputs, which can have multiple beneficial effects: 1) at the agroecological level, contributing to the maintenance of the ecological equilibrium soil–plant‐microbiota, 2) also on the economic side, making agriculture less dependent on costly chemical inputs, which is particularly important for smallholder farmers and, 3) furthermore, through seed priming's ability to trigger preemptive plant defense mechanisms against pest attacks and diseases. It contributes to safer and healthier agricultural food products that meet consumers' demands.

It is therefore imperative to conduct further research to fully elucidate the molecular mechanisms by which the different seed priming treatments exert their effects, and how these are related to the specific priming strategy. Moreover, the potential of seed priming to induce transgenerational epigenetic effects that confer protection to the progeny, going beyond the boundaries of genetic selection, is particularly fascinating. The multiple economic, ecological, and adaptive benefits underscore the potential of seed priming as a transformative tool in the pursuit of resilient and sustainable crop production in the face of increasing environmental pressures.

## AUTHOR CONTRIBUTIONS

VF, RB, MGG and VA: conceptualization. EC and LG: wrote first draft. EC, VA and MGG: wrote and revised subsequent versions. EC and BOG: prepared figures and tables. All authors revised and edited the manuscript. All authors approved the final version of the manuscript.

## Data Availability

Non‐applicable.
